# A comparison of radiological descriptions of spinal cord compression with quantitative measures, and their role in non-specialist clinical management

**DOI:** 10.1371/journal.pone.0219380

**Published:** 2019-07-22

**Authors:** Jennifer Tempest-Mitchell, Bryn Hilton, Benjamin M. Davies, Aria Nouri, Peter J. Hutchinson, Daniel J. Scoffings, Richard J. Mannion, Rikin Trivedi, Ivan Timofeev, John R. Crawford, Douglas Hay, Rodney J. Laing, Mark R. N. Kotter

**Affiliations:** 1 School of Clinical Medicine, University of Cambridge, Cambridge, United Kingdom; 2 Division of Neurosurgery, Department of Clinical Neurosciences, University of Cambridge, Cambridge, United Kingdom; 3 Division of Neurosurgery, Toronto Western Hospital, University Health Network, Toronto, Ontario, Canada; 4 Department of Radiology, Cambridge University Hospitals NHS Foundation Trust, Cambridge, United Kingdom; 5 Anne McLaren Laboratory for Regenerative Medicine, Welcome Trust MRC Cambridge Stem Cell Institute, University of Cambridge, Cambridge, United Kingdom; George Washington University, UNITED STATES

## Abstract

**Introduction:**

Magnetic resonance imaging (MRI) is gold-standard for investigating Degenerative Cervical Myelopathy (DCM), a disabling disease triggered by compression of the spinal cord following degenerative changes of adjacent structures. Quantifiable compression correlates poorly with disease and language describing compression in radiological reports is un-standardised.

**Study design:**

Retrospective chart review.

**Objectives:**

1) Identify terminology in radiological reporting of cord compression and elucidate relationships between language and quantitative measures 2) Evaluate language’s ability to distinguish myelopathic from asymptomatic compression 3) Explore correlations between quantitative or qualitative features and symptom severity 4) Investigate the influence of quantitative and qualitative measures on surgical referrals.

**Methods:**

From all cervical spine MRIs conducted during one year at a tertiary centre (N = 1123), 166 patients had reported cord compression. For each spinal level deemed compressed by radiologists (N = 218), four quantitative measurements were calculated: ‘Maximum Canal Compromise (MCC); ‘Maximum Spinal Cord Compression’ (MSCC); ‘Spinal Canal Occupation Ratio’ (SCOR) and ‘Compression Ratio’ (CR). These were compared to associated radiological reporting terminology.

**Results:**

1) Terminology in radiological reports was varied. Objective measures of compromise correlated poorly with language. “Compressed” was used for more severe cord compromise as measured by MCC (p<0.001), MSCC (p<0.001), and CR (p = 0.002).

2) Greater compromise was seen in cords with a myelopathy diagnosis across MCC (*p*<0.001); MSCC (*p* = 0.002) and CR (*p*<0.001). “Compress” (p<0.001) and “Flatten” (p<0.001) were used more commonly for myelopathy-diagnosis levels.

3) Measurements of cord compromise (MCC: p = 0.304; MSCC: p = 0.217; SCOR: p = 0.503; CR: p = 0.256) and descriptive terms (p = 0.591) did not correlate with i-mJOA score.

4) The only variables affecting spinal surgery referral were increased MSCC (p = 0.001) and use of ‘Compressed’ (p = 0.045).

**Conclusions:**

Radiological reporting in DCM is variable and language is not fully predictive of the degree of quantitative cord compression. Additionally, terminology may influence surgical referrals.

## Introduction

‘Degenerative Cervical Myelopathy’(DCM) refers to spinal cord disease triggered by degeneration of the cervical spine, including cervical spondylotic myelopathy, degenerative disc disease, ossification of the posterior longitudinal ligament and ossification of the ligamentum flavum [1]. DCM is the most common form of spinal cord dysfunction [2,3] with treatment limited to surgical decompression. Surgery can prevent further injury and improve neurological function and general health [4]. However, many patients retain life-long disabilities and reduced quality of life [4,5]. Prompt diagnosis and treatment are key to preserving function and ensuring good recovery [6], however diagnosis is frequently delayed [1,7]. As treatment is limited to surgical decompression, new international guidelines recommend that all patients with DCM are reviewed by specialists who can offer surgery [8]. Initial assessment and diagnosis, however, is typically carried out by other specialities.

Diagnosis of DCM requires clinical signs and symptoms, confirmed with MRI (magnetic resonance imagining) examination. MRI [9] is the best imaging modality for assessing extent of cord compromise or injury [10] and typical features include visible cord compression, altered cord signal intensity, canal stenosis, altered sagittal spinal alignment and ligamentous changes [11].

Despite investigation, no standard MRI features consistently representing disease severity in DCM have been found [12], and whilst cord compression is considered a hallmark, its extent correlates poorly with severity. This may be due to dynamic injury mechanisms undetected by standard MRI protocols [13] or biological differences in responses to mechanical stress. Significant cord compression can be present in asymptomatic individuals [14,15].

Currently, various quantitative measurements of cord compromise have been described, including ‘Transverse Area’, ‘Compression Ratio’, ‘Maximum Canal Compromise’, ‘Maximum Spinal Cord Compression’ and ‘Spinal Cord Occupation Ratio’. However, their chief usage is in research. Whilst such measurements provide objective and quantitative measures of cord compromise, in daily practice, non-specialist clinicians are primarily informed by the qualitative reports provided with MRIs. Whilst standardised nomenclature for MRI features such as disc pathology is well-established, no such guidance exists for radiologists reporting cervical spine imaging. Semi-quantitative and qualitative scales for cervical stenosis exist [16] but are not widely used. We hypothesised that language used by radiologists reporting cord compromise influences clinical management.

This study aimed to 1) identify terminology used in radiological reporting of spinal cord compromise 2) compare this with objective, quantitative measures of cord compromise 3) evaluate its ability to distinguish myelopathic from asymptomatic cord compromise and 4) investigate whether language influences referral to spinal surgeons.

## Methods

This was a retrospective study examining data from all patients receiving a cervical MRI in one year at a tertiary NHS centre (N = 1123).

Author BH assessed radiological reports for each MRI for reference to spinal cord involvement. For each patient in whom compromise was identified (N = 166), the main descriptive term used to describe the cord at the affected level (e.g. flattened), as well as any qualifiers (e.g. mild) were recorded. In patients with multiple levels of cord involvement suggested, details for each were recorded and analysed separately, giving 218 unique cord levels.

Radiological reports were primarily authored by consultant neuroradiologists, but a minority (N = 9) were written by radiology trainees and endorsed by supervising consultants. In the single case where the consultant used a different term to the trainee, this was recorded in preference.

For each spinal level, four ratio measurements were calculated using raw measurements gathered from computer-based MRI records: ‘Maximum Canal Compromise (MCC) [17]; ‘Maximum Spinal Cord Compression’ (MSCC) [17]; ‘Spinal Cord Occupation Ratio ‘(SCOR) [11, 18] and ‘Compression Ratio’ (CR) [19–21] (Table 1). Drawn from previous literature, these represent the selection felt to best reflect visible compromise and offer clinical significance [11]. Greater cord compromise is indicated by a larger MCC, MSCC or SCOR, or smaller CR. Reflecting work by Nouri et al. [22] we defined an SCOR value of ≥70% as diagnostic of congenital stenosis (or cord-canal mismatch). Ratio measurements were chosen to allow for better standardisation and comparison between patients than raw measurements of cord diameter. The accuracy of raw measurements (used to calculate ratio values) was verified by second researcher BD. Bland-Altman analysis showed acceptable agreement (SD = 0.24).

**Table 1:** A summary of the key features of the four quantitative measures of compression calculated.

Measurement	Description	Formula	Reliability
MCC	Ratio of the midsagittal diameter of the spinal canal at the compression site divided by the average diameter of the spinal canal at the closest non-compressed regions above & below	$\frac{Midsagital\;AP\;canal\;diameter}{(Midsagital\;AP\;diameter\;first\;normal\;level\;aove+first\;normal\;level\;below)/2}$	Intra- and inter- observer ICCs reported previously as 0.88 ± 0.1and 0.75 ± 0.04 for T1 images [23]
MSCC	Ratio of the midsagittal diameter of the spinal cord at the compression site divided by the average diameter of the spinal cord at the closest non-compressed regions above & below	$\frac{Midsagital\;AP\;cord\;diameter}{(Midsagital\;AP\;diameter\;first\;normal\;level\;aove+first\;normal\;level\;below)/2}$	Intra- and inter- observer ICCs reported previously as 0.76 ± 0.08 and 0.79 ± 0.09 for T2 images [23]
SCOR	Ratio of the sum of the cord width above and below, and the sum of the canal width above and below the point of compression	$\frac{AP\;Cord\;diameter\;above\;compression+Cord\;diameter\;below\;compression}{AP\;Canal\;diameter\;above\;compression+Canal\;diameter\;below\;compression}$	Unknown
CR	The ratio of the sagittal diameter divided by the transverse diameter of the spinal cord observed on axial T1WI	$\frac{Sagittal\;cord\;diameter\;at\;level\;of\;compression}{Transvere\;cord\;diameter\;at\;level\;of\;compression}$	Intra- and inter- observer ICCs reported previously as 0.82 ± 0.13 and 0.80 ± 0.05 on axial T2 images [23]

*MCC = Maximum Canal Compromise*, *MSCC = Maximum Spinal Cord Compression*, *SCOR = Spinal Cord Occupation Ratio*, *CR = Compression Ratio*, *T1WI = T1 Weighted Imaging*, *AP = Anteroposterior*, *ICC = Intraclass correlation*

**Fig 1:**
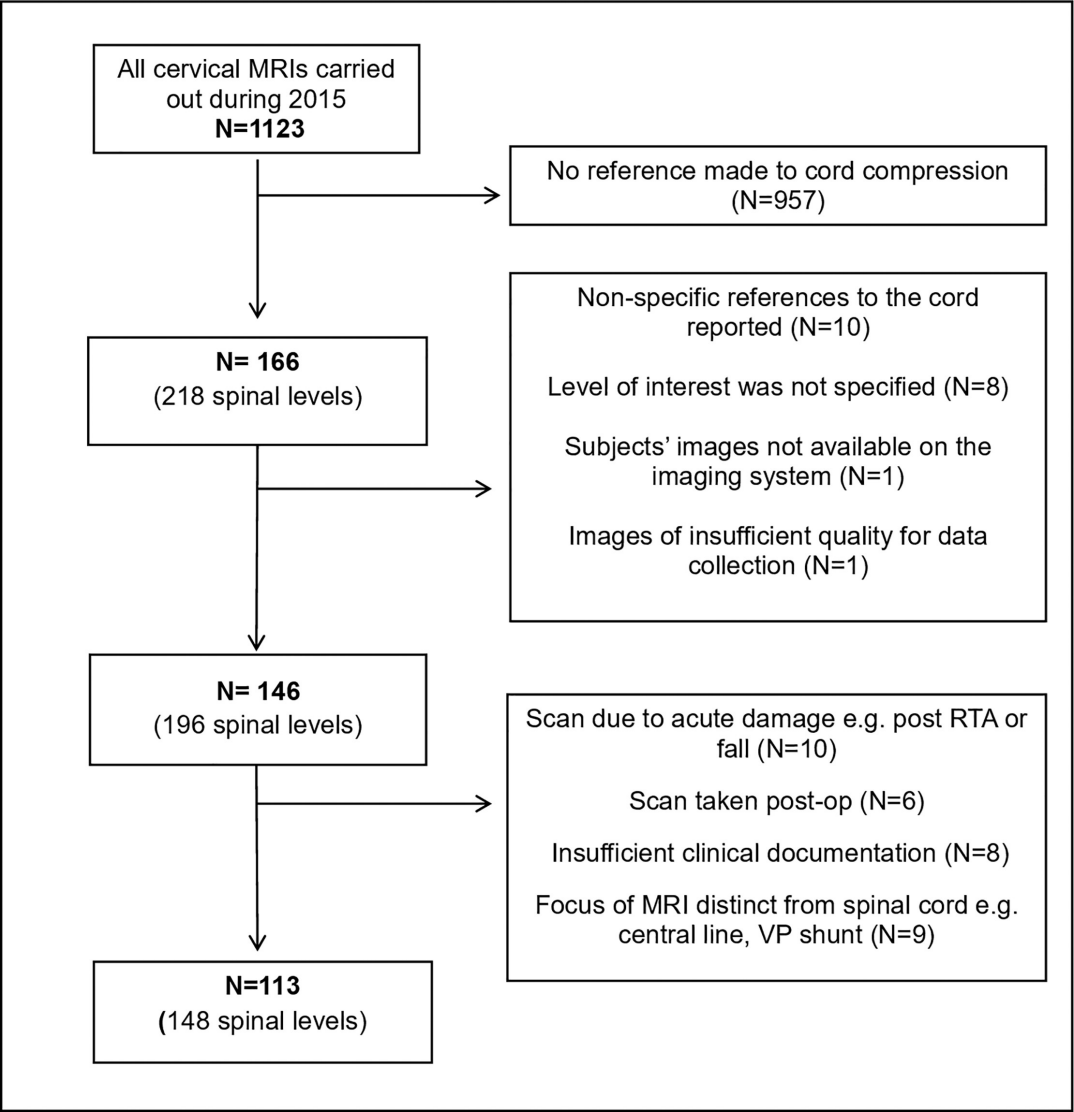
A diagram outlining the stages of exclusion criteria for subjects. *Key*: *MRI = Magnetic Resonance Imaging*, *RTA = Road Traffic Accident*, *VP = Ventriculoperitoneal*.

Data summarising the treatment pathway of this cohort were extracted from the hospital database of patient records. Here, patients presenting acutely, with non-degenerative conditions (e.g. malignancy), or for whom insufficient documentation existed, were excluded, leaving 113 unique patients, and 148 spinal levels overall (Fig 1).

The mJOA is the most common assessment of DCM [24], however it relies upon clinicians completing specific assessments. Symptom severity was therefore assessed using clinical notes, and quantified with the i-mJOA, a modified form of the mJOA [25] developed and validated by our team [26].

### Statistical methods

Statistical analysis was carried out using SPSS (v24, UK), with statistical significance at the level of p<0.05. Descriptive statistics were generated for all data sets and each was assessed for normal distribution as required, using Shapiro-Wilk and visual inspection.

To assess whether a relationship existed between the quantitative degree of cord compromise at each spinal level and the phrase describing that level in the associated radiological report, one-way ANOVA (and post-hoc Tukey’s range test)—or in cases of unequal variances, Welch’s test (and post-hoc Games-Howell analysis)—was used to compare the main descriptive terms against MCC, MSCC and CR. Only descriptive terms used ten or more times were included in analysis.

To compare characteristics of cords associated with a myelopathy diagnosis to those with no such diagnosis, it was assumed the diagnosis was applicable to all levels with compromise. Comparison of quantitative measures of compromise between myelopathy-diagnosis and non-myelopathic spinal cords was carried out using one-way ANOVA for MCC, MSCC and CR and chi-squared testing to compare myelopathy rates in levels with a SCOR of more or less than 70%. Use of main descriptive terms and qualifiers in myelopathic and non-myelopathic cords was evaluated using Chi-Squared testing, and post-hoc analysis with adjusted p-values.

Binary logistic regression models were used to consider the significance of quantitative measures of cord compromise and descriptive phrase on predicting whether spinal levels were referred to spinal surgeons.

## Results

### Subject characteristics

Data from 113 patients was analysed. 46% of subjects were male, with a mean age of 55.1 years and 32% (N = 36) were at any time diagnosed with myelopathy. Patient characteristics are presented in Table 2.

**Table 2:** A table summarising the characteristics of subjects included in the study.

*General Characteristics*	
Age ± SD, (Range)	55.1 ± 14.0 (21–91)
Male, n (%)	52 (46)
***MRI Features***	
Mean MCC ± SD (Range)	25.7 ± 14.6 (0.8–62.8)
Mean MSCC ± SD (Range)	17.4 ± 13.8 (0.0–63.1)
Mean SCOR ± SD (Range)	61.3 ± 8.7 (40.8–109.2)
Mean CR ± SD (Range)	0.42 ± 0.11 (0.16–0.71)
***Clinical Pathway***	
Patients diagnosed with myelopathy, n (%)	36 (32)
Patients ultimately reviewed by a spinal surgeon, n (%)	81 (72)
Mean mJOA on assessment by spinal surgeon ± SD, (Range)	15.4 ± 1.8 (9–18)
Patients offered surgical treatment, n (%)	34 (30)
Mean time to surgery from assessment (months) ± SD (Range)	3.6 ± 3.7 (0–18)

*SD = Standard Deviation*, *MRI = Magnetic Resonance Imaging*, *MCC = Maximum Canal Compromise*, *MSCC = Maximum Spinal Cord Compression*, *SCOR = Spinal Cord Occupation Ratio*, *CR = Compression Ratio*, *mJOA = Modified Japanese Orthopaedic Association Scale*

### What is the relationship of qualitative terms with quantitative measures?

A range of vocabulary was used to describe spinal cord levels. Within the cohort, 11 distinct descriptive terms and 11 qualifier terms were identified in radiological reports, though 52% of reports used no qualifiers. Details are summarised in Tables 3 and 4. Only descriptive terms used ten or more times (‘Compress’, ‘Indent’, ‘Abut’, ‘Flatten’ and ‘Touch’) were included in statistical analysis.

**Table 3:** Mean values of each of the four measures of compression for each of the main descriptive terms used in radiological reports.

Descriptive Phrase	N	Mean MCC ± S.D	Mean MSCC ± S.D	Mean SCOR ± S.D	Mean CR ± S.D
‘Compress’	42	39.8±15.6	26.2±8.7	63.9±8.7	0.37±0.12
‘Indent’	40	17.6±13.2	16.0±11.2	59.4±11.2	0.43±-0.10
‘Abut’	28	20.0±9.0	9.9±6.7	58.8±6.7	0.48±0.07
‘Flatten’	11	25.8±16.4	20.5±6.2	65.2±6.2	0.40±0.14
‘Touch’	10	19.8±6.9	11.1±8.2	60.1±8.2	0.46±0.10
‘Mould’	6	18.3±7.6	10.3±3.8	62.9±3.8	0.48±0.04
‘Encroach’	5	23.6±4.5	10.4±5.1	59.9±5.1	0.43±0.11
‘Distort’	3	32.2±9.0	19.8±9.4	61.1±9.4	0.36±0.12
‘Compromise’	1	13.4	11.9	63.1	0.40
‘Contact’	1	20.0	19.4	63.2	0.43
‘Displace’	1	23.6	13.8	64.4	0.38

*SD = Standard Deviation*, *MCC = Maximum Canal Compromise*, *MSCC = Maximum Spinal Cord Compression*, *SCOR = Spinal Cord Occupation Ratio*, *CR = Compression Ratio*

**Table 4:** Mean values of each of the four measures of compression for each of the combinations of qualifier term and description used in radiological reports.

Qualifier Term	Main Descriptive Term	N	Mean MCC	Mean MSCC	Mean SCOR	Mean CR
Greater	Compromise	1	13.37	11.86	63.10	0.40
Just	Abut	8	16.05	5.61	57.58	0.51
	Encroach	4	17.12	9.85	60.46	0.47
Lesser Degree	Compression	2	36.95	21.71	57.98	0.28
Mild	Compression	20	38.30	24.44	62.53	0.40
	Distortion	1	40.78	29.77	63.59	0.49
	Flatten	2	19.17	24.01	66.16	0.42
	Indent	22	18.47	15.94	59.64	0.44
Mild-Moderate	Compression	1	57.73	28.70	59.28	0.25
Minimal	Indent	2	17.07	13.72	64.79	0.39
Minor	Mould	2	21.29	3.78	59.74	0.51
Moderate	Compression	2	46.02	40.64	67.88	0.40
Moderate-Marked	Compression	1	43.68	25.76	75.86	0.39
None	Abut	20	21.54	11.68	59.26	0.47
	Compression	14	41.07	26.81	66.57	0.32
	Contact	1	20.00	19.42	63.18	0.43
	Displace	1	23.56	13.82	64.40	0.38
	Distortion	1	47.91	17.43	50.70	0.27
	Encroach	1	49.51	12.61	57.77	0.26
	Flatten	9	23.98	18.54	64.81	0.41
	Indent	16	16.40	16.47	58.43	0.42
	Mould	4	16.85	13.56	64.49	0.46
	Touch	10	19.78	11.13	60.12	0.46
Slight	Compression	2	32.00	28.34	57.32	0.55
	Distortion	1	7.88	12.28	69.09	0.33

*MCC = Maximum Canal Compromise*, *MSCC = Maximum Spinal Cord Compression*, *SCOR = Spinal Cord Occupation Ratio*, *CR = Compression Ratio*

#### Maximum canal compromise (MCC)

**Fig 2:**
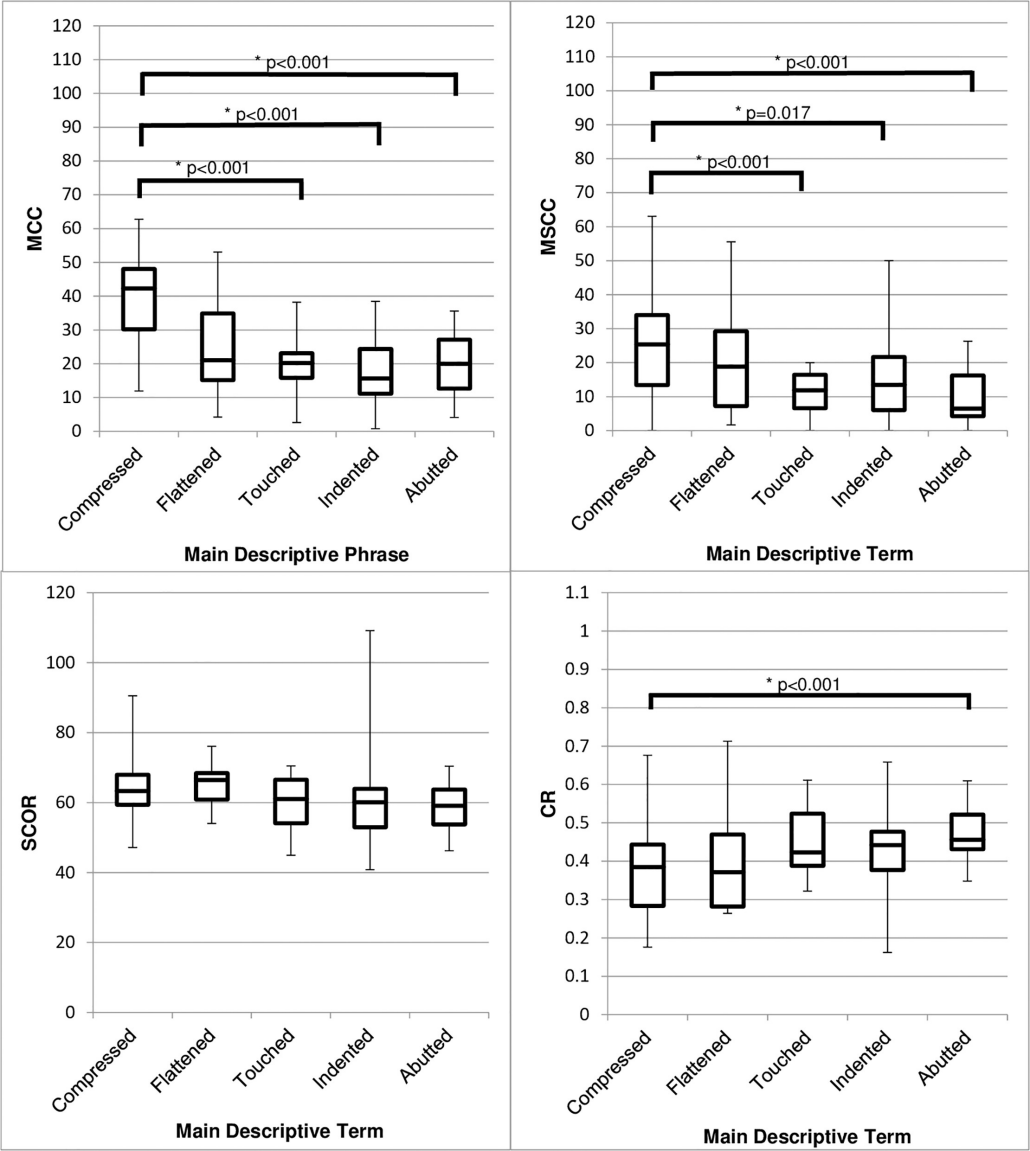
Box and whisker diagrams showing the range, IQR, and median values of quantitative cord compression for the five most common terms used in radiological reports to describe spinal cord involvement. Four different measures of compression are shown: MCC, MSCC, SCOR and CR. A greater MCC, MSCC and SCOR or lower CR indicates greater compression (N = 131). *Key*: *IQR = Interquartile range*, *MCC = Maximum Canal Compromise*, *MSCC = Maximum Spinal Cord Compression*, *SCOR = Spinal Cord Occupation Ratio*, *CR = Compression Ratio*.

Comparing main descriptive term to the ratio measurements showed a significant difference in MCC between groups (Welch’s test, (p<0.001)). MCC was higher in the group of MRIs where cords were described as ‘Compressed’ compared to ‘Abutted’ (19.9 ± 7.6, p<0.001), ‘Indented’ (22.3 ± 7.1, p<0.001) or ‘Touched’ (20. 0± 10.9 p<0.001). There were no other significant differences between groups (Fig 2).

#### Maximum spinal cord compression (MSCC)

MSCC differed significantly between groups (Welch’s test (p<0.001)) and was higher in the group of cords described as ‘Compressed’ compared to ‘Abutted’ (16.2±8.0, p<0.001), ‘Indented’ (10.2±8.9, p = 0.017) or ‘Touched’ (15.1±9.3, p<0.001). (Fig 2).

#### Compression ratio (CR)

Comparing main descriptive term and CR, showed a significant difference between groups (Welch’s test, (p = 0.002)), with CR lower in the group of cords described as ‘Compressed’ compared to ‘Abutted’ (-0.11±0.06, p<0.001), indicating greater compromise in the former. There were no other significant differences between groups (Fig 2).

Overall, objective measures of cord compromise correlated poorly with radiological descriptions. The term “Compressed” seemed to be used in more severe cord compromise as measured by MCC, MSCC, and CR.

A wide combination of qualifier terms was used to modify descriptive terminology (Table 4), however the sample size was insufficient to allow analysis of the relationship between qualifiers and degree of cord compromise.

### Do qualitative or quantitative features identify myelopathic spinal cord levels?

Within the sample, 48 spinal levels (32.3%) identified by radiologist were associated with a diagnosis of DCM. Within this sample, patients showing compression at more than one site were excluded from analysis, as it is not possible to pinpoint which level was responsible for their symptoms. This left 27 unique spinal levels associated with a diagnosis of DCM.

**Fig 3:**
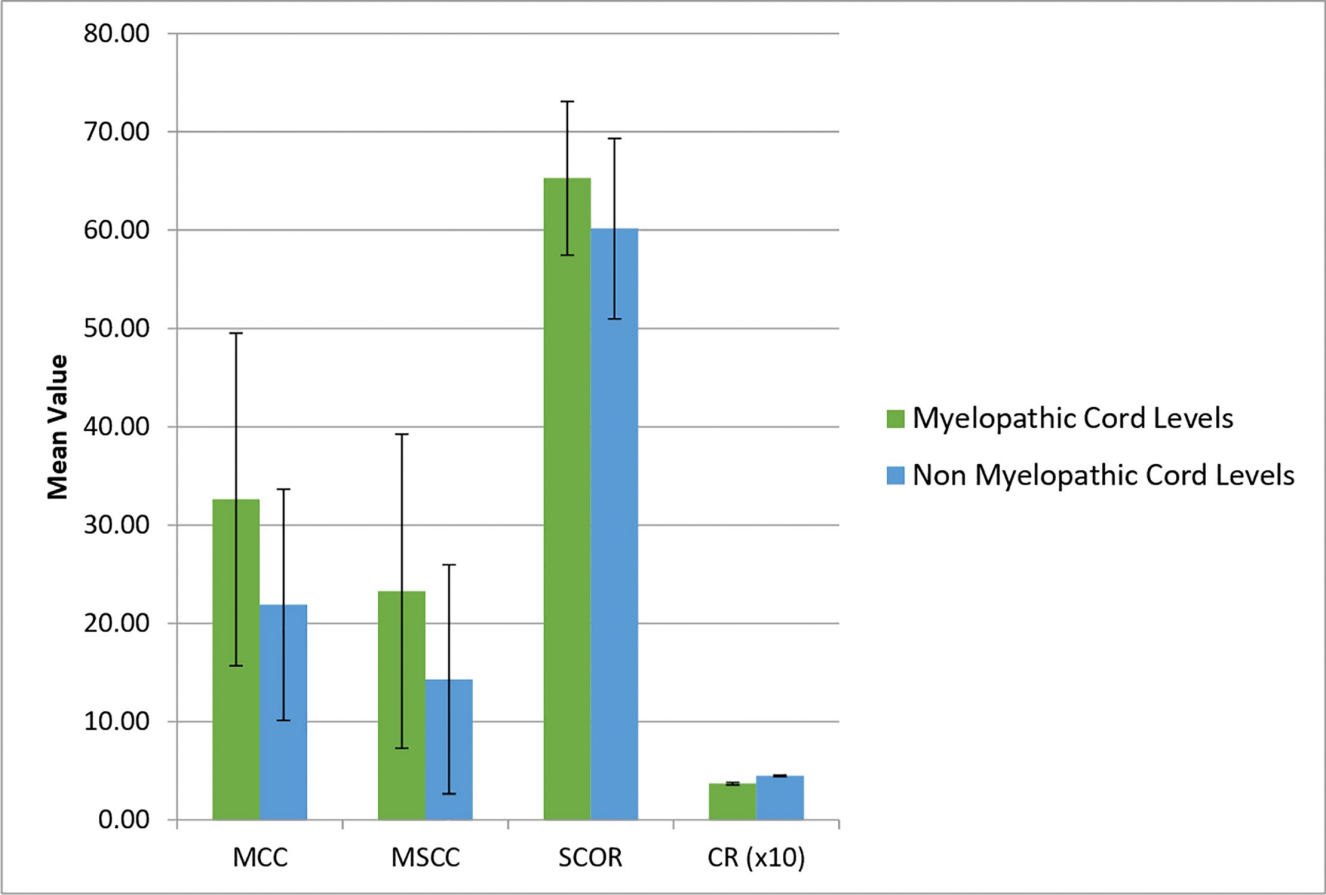
A graph showing the mean value of four measures of compression in myelopathic and non-myelopathic spinal cord levels. Error bars show standard deviation of compression (N = 127). *Key*: *MCC = Maximum Canal Compromise*, *MSCC = Maximum Spinal Cord Compression*, *SCOR = Spinal Cord Occupation Ratio*, *CR = Compression Ratio*.

Comparing cord levels in patients considered myelopathic to those without the diagnosis found significantly greater compromise in myelopathy-diagnosis cords across three quantitative measurements (MCC: *p* <0.001; MSCC: *p* = 0.002 and CR: *p*<0.001) (Fig 3). Rates of myelopathy diagnosis also differed between cords with an SCOR greater than 70% and an SCOR less than 70% (χ^2^ = 4.211, p = 0.040).

**Fig 4:**
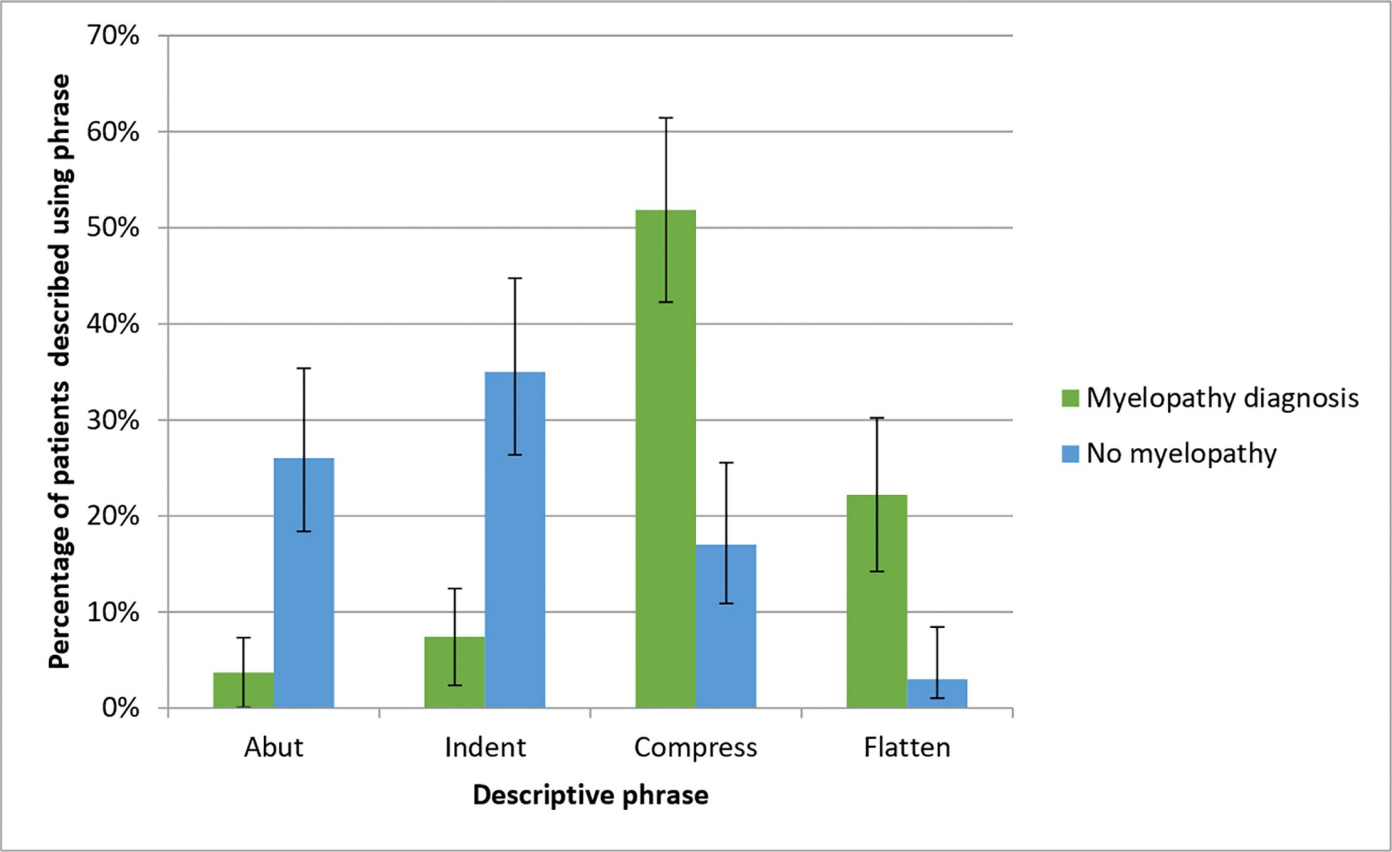
A graph showing the percentage of spinal cord levels with and without a diagnosis of myelopathy described with each of the four most common descriptive terms. Error bars represent 95% confidence interval (N = 104).

Considering the term used to describe myelopathic and non-myelopathic cord levels (Table 5), analysis of the four most popular terms (‘Abut’, ‘Indent’, ‘Compress’ and ‘Flatten’) showed a significant difference in the pattern of descriptive terms between the two groups (χ(2) = 31.242, *p* = <0.001). Post-hoc analysis revealed that ‘Compress’ (p<0.001) and ‘Flatten’ (p<0.001) were used more often to describe myelopathy-diagnosis cord levels, whilst ‘Indent’ (p = 0.002) more often described levels without the disease (Fig 4).

**Table 5:** Number of spinal levels described with each main descriptive term for the cords of patients with and without myelopathy.

	Compress	Indent	Abut	Flatten	Touch	Mould	Encroach	Distortion	Contact	Displace	Compromise
**Myelopathy Diagnosis**	25	5	2	8	3	0	2	2	0	0	1
**No Diagnosis**	17	35	26	3	7	6	3	1	1	1	0

### Do qualitative or qualitative features correlate with symptom severity?

Data was available from all 81 patients receiving a surgical consultation to calculate an i-mJOA score [26], an 18 point, semi-quantitative assessment scale of disease severity, based on the mJOA score [27]. Individuals with cord compromise but no evidence of myelopathy receive an i-mJOA score of 18.

Pearson product-moment-correlation analysis showed no correlation between the four ratio measurements (MCC (r = -0.116, p = 0.304), MSCC (r = -0.139, p = 0.217), SCOR (r = -0.076, p = 0.503), CR (r = 0.128, p = 0.256)) and i-MJOA at the time of first appointment with a spinal surgeon.

Similarly, one-way ANOVA showed no relationship between descriptive term used and i-mJOA at the time of first appointment with a spinal surgeon (p = 0.591).

Restricting analysis to the 27 spinal levels associated with a myelopathy diagnosis also showed no relationship between i-mJOA and descriptive term (p = 0.254), MCC (r = -0.215, p = 0.324), MSCC (r = -0.064, p = 0.771), SCOR (r = -0.383, p = 0.071) or CR (r = -0.248, p = 0.254).

Overall, objective measurements of cord compromise do not appear to correlate with severity of DCM symptoms.

### Do quantitative or qualitative features of DCM predict surgical consultation?

Within the cohort, 81 patients (72%) with 107 spinal levels (73%) were assessed by spinal surgeons. Of these patients, 44 (54%) received a diagnosis of myelopathy.

**Fig 5:**
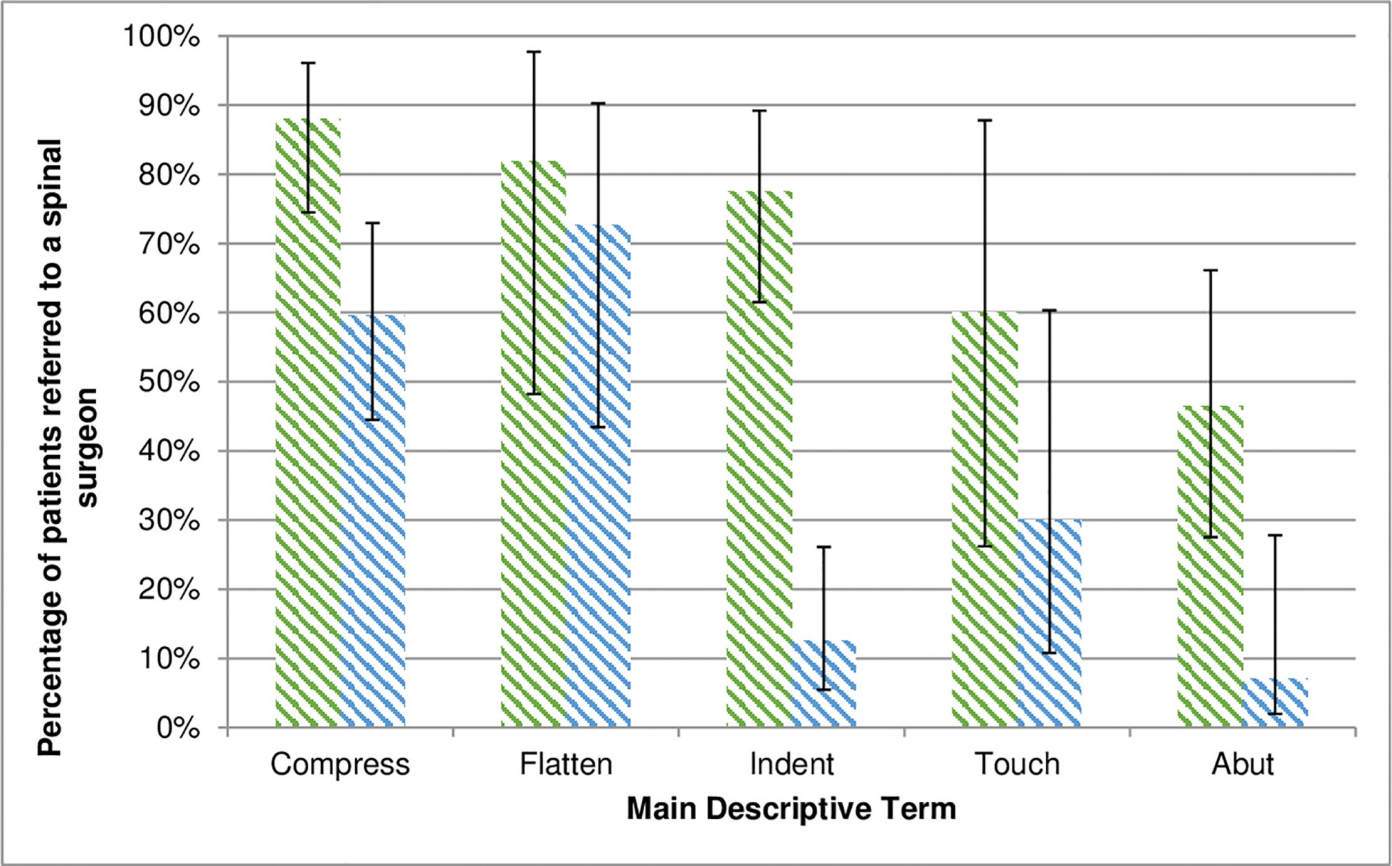
A graph showing the percentage of patient spinal cord levels seen by a surgeon for each main descriptive term. Error bars represent 95% confidence interval.

The percentage of cord levels reviewed by spinal surgeons differed across the four most common descriptive terms (‘Abut’, ‘Compress’, ‘Flatten’, ‘Indent’ and ‘Touch’) used in radiological reports (N = 131, Χ^2^ = 16.7, p = 0.002) with more cords described as ‘Compressed’ (p<0.001), and fewer described as ‘Abutted’ (p<0.001) referred to spinal surgeons (Fig 5). Supporting this, a binary logistic regression model considering choice of descriptive phrase (χ2 = 16.72, p = 0.005) found a model correctly classifying 73.6% of cases, where use of ‘Compressed’ was the only significant variable predicting referral (p = 0.045).

Two of the four quantitative measures of cord compromise were significantly different in those spinal levels referred to surgeons compared to those which were not. MCC (p = 0.009) and MSCC (p<0.001) showed greater compromise in patients referred for surgical review, however neither SCOR (p = 0.220) nor CR (p = 0.063) varied. A binary logistic regression model considering MCC, MSCC, CR and SCOR of greater than 70% found a model (χ2 = 18.46, p = 0.001) correctly classifying 76.4% of cases, in which the only variable significantly affecting referral was increased MSCC (p = 0.001).

## Discussion

Our study showed that many different terms are used in radiological reporting of spinal cord involvement, however their quantitative features overlap greatly. Whilst some relationships were identified- for example, the qualitative term 'Compressed' was associated with greater quantifiable compromise- this was inconsistent. Moreover, neither qualitative or quantitative measures of cord involvement correlated with clinical symptoms, despite compression acting as a determinant of referral to spinal surgeons.

These preliminary results raise several key issues: firstly, that little relationship was seen between quantitative and qualitative features of spinal MRIs; secondly, the apparent ability of radiological reporting to influence clinicians, and finally, the requirement for MRI to diagnose DCM despite its inability to specify or stage disease.

Debate over the value of qualitative versus quantitative descriptors in radiology is longstanding and has been considered across various conditions [28–30]. Typically, such work compares the relative benefit of quantitative measurements to qualitative grading systems with set descriptive criteria and a proven inter/intra-rater reliability. In DCM, however, description of cord compromise uses terms chosen at a radiologist’s discretion, with no clear guidance as to their individual meaning. This is particularly important when we know radiological reports are influential in clinical decision-making. Surveys suggests that the majority of clinicians rely on radiological reports to guide their practice, and believe that radiologists are equally or better able to interpret imaging than themselves [31]. Additionally, evidence suggests that radiologists’ choice of language may be confusing to other clinicians and affect clinical practice. For example, work has suggested that use of the word ‘infiltrate’ in reports produces a range of non-overlapping interpretations by clinicians regarding possible underlying pathology and diagnoses [32]. Equally, reporting of chest radiographs has been shown to affect both diagnosis and management in childhood respiratory disease [32–34]. We also know that there is mismatch between how radiologists and requesting clinicians would like reports to be made [31]. Collectively, this literature suggests that radiologists’ choice of language may have unintended effects on patient care. This is consistent with our findings suggesting that language choice may influence non-expert clinician’s decision whether to refer patients with DCM.

This becomes particularly significant given the delays patients face in diagnosis: typically over 2 years [7], delaying treatment. The chance of full recovery is greatest if surgery is offered within 6 months of symptom onset [35] and cervical MRIs are key in the diagnostic pathway [36]. Additionally, even patients with demonstrable cord compression (but no myelopathy) risk developing DCM, and may sometimes opt to undergo surgery [37]. Therefore, it is recommended that all patients with MRI features and symptoms of DCM are assessed by spinal surgeons [38].

The route to a diagnosis of myelopathy involves multiple different specialities [39] with a shared key diagnostic stage in MR imaging. MRI is therefore an attractive target for strategies to improve patient care. The question remains however, how to improve interpretation by non-specialist clinicians.

Quantitative measurements are attractive in their objectivity, however, as demonstrated, their relationship to myelopathy is inconsistent: some quantitative MRI measures of compromise may relate to clinical symptoms of DCM [40] but, overall, degree of radiological compromise correlates poorly with disease severity. Patients with cord compression may not suffer from myelopathy [41] and some patients suffer myelopathy without visualised compression due to dynamic injury [13].

There are of course extensive advancements in MR imaging [42] and techniques such as fractional anisotropy have the potential to detect microstructural changes [43] which better correlate with severity [44], and may also detect subclinical spinal cord injury [45]. However, these techniques are far removed from current practice.

Consequently, isolated MR imaging cannot currently replace clinical assessment and it is notable that interpretation of MRI reports by non-expert clinicians may contribute to false reassurances and variable care. To prevent confusion for non-expert clinicians, descriptive terminology could be removed from reporting and replaced by statements of consistency (or non-consistency) with DCM but further investigation is needed to confirm the value of such an approach. There are, of course, limitations to the conclusions of this study- data was drawn retrospectively from a single centre, and patterns of language likely differ across individuals, centres and countries. Furthermore, we focussed on the quantifiable degree of cord compromise visible on MRI without considering other features which could influence radiologists’ choice of language (for example signal hyperintensity [46]) or other clinical factors contributing to seeking a spinal surgery consult. However, while choices of wording may differ, the subjectivity implicit with the use of qualitative descriptions identified here is likely to be present elsewhere. Moreover, the relationship with myelopathy diagnosis and severity is in-keeping with the wider medical literature [1].

## Conclusions

This is the first study considering radiological reporting and routine patient care in DCM. Many different terms are used to describe spinal cord involvement and choice of word does not consistently represent quantitative compromise. Moreover, objective assessments of spinal cord compromise do not correlate with severity of myelopathy symptoms. However, choice of qualitative description may influence decisions regarding referral to spinal surgery and as all symptomatic patients are now recommended to receive assessment by a spinal surgeon [8] it is possible that ambiguities in language are affecting patients’ treatment and quality of life. Any evidence of spinal cord compression with myelopathy symptoms should therefore be considered significant.

## Supporting information

S1 Table Table of raw data. (XLSX)
